# Authors’ reply

**Published:** 2010

**Authors:** Zakir M Shaik, M Shukla, Simi Zaka-ur-rab, J Ahmad, Sajid Mahmood

**Affiliations:** Retina Service, Institute of Ophthalmology, Jawaharlal Nehru Medical College, Aligarh Muslim University, Aligarh, India; 1 Division of Endocrinology, Department of Medicine, Jawaharlal Nehru Medical College, Aligarh Muslim University, Aligarh, India

Dear Editor,

We thank Chalisgoankar *et al*.,[1] for having shown interest in our article.[2] Our point wise reply to their comments is as under:

1. We agree that number of cases and controls was rather less. One of the reasons was that the study was conducted over a short period (21 months) during which we could select only 23 cases and 12 age and sex-matched controls who fulfilled all the requisite inclusion criteria. Patients receiving corticosteroids in any form (including traditional or alternative medicines which might contain some form of steroids) within last one month or with other conditions which could independently alter endogenous cortisol levels were excluded.
2. Though it appears reasonable to assume that ″endogenous cortisol may not act pathologically unless it is outside normal range″, our study has documented an interesting finding that pathological changes of idiopathic central serous chorioretinopathy (ICSC) occurred even when cortisol levels were within normal range. It, however, clearly showed that mean serum cortisol levels were significantly higher (but not higher than normal) in patients with ICSC as compared to controls. Whereas 12 (52.17%) cases had levels > 500 nmol/L, all controls had lower levels. Moreover, to the best of our knowledge, ours is the only study where serum cortisol levels were tabulated for each and every case.
3. Morning cortisol levels were estimated since cortisol levels follow a relatively predictable circadian rhythm with an early morning peak after awakening, a rapid decrease over the next few hours, followed by a more gradual decline over the course of the day to very low levels by bedtime.[3] A single estimation of the 08:00 hour serum cortisol reflects endogenous activity of the hypothalamo–pituitary–adrenal axis.[4]
4. The learned authors have mentioned some interesting observations of their own study. However, since we are not aware of their exact methodological details as well as criteria for exclusion, it would not be appropriate to either compare the two studies or comment on the findings of their study. It may be worthwhile to mention that many a times patients first seek treatment from local practitioners (including untrained traditional or alternative medicine practitioners) who invariably prescribe steroids in some form. Exogenous steroid intake in any form leads to suppression of the hypothalamo–pituitary–adrenal axis resulting in lowering of endogenous serum cortisol for variable periods of time.[5]
5. Even as the precise mechanism of steroids in the etiopathogenesis of ICSC has not been established, some of their effects on choriocapillaries and retinal pigment epithelium (RPE) have been mentioned in various studies. Glucocorticoids are known to affect choroidal circulation,[6] inhibit collagen formation (a main component of Bruch’s membrane), alter ion and water transport of epithelia. Cortisol may also directly damage the RPE cells or their tight junctions and may delay any reparative process by suppressing the synthesis of extracellular matrix components and inhibiting fibroblastic activity.[7]
6. We do strongly feel that more, preferably larger studies on endocrinological association of ICSC are needed in order to have a better understanding of this important ophthalmological problem.
